# Surgical management of middle ear cholesteatoma and reconstruction at the same time

**Published:** 2014-09-30

**Authors:** Pedro Blanco, Francisco González, Jorge Holguín, Claudia Guerra

**Affiliations:** 1 Professor. Specialization in Otology and Neuro-Otology, University of Valle. Cali, Colombia; 2 Otology and Neuro-Otology, University of Valle. Cali, Colombia

**Keywords:** Cholesteatoma, ossiculoplasty, canal-wall-down mastoidectomy (CWD), canal-wall-up mastoidectomy (CWU), reconstruction mastoidectomy, COG

## Abstract

**Introduction:**

In the surgical management of cholesteatoma, one can opt for a closed technique (simple mastoidectomy) or open surgery (radical mastoidectomy). Open mastoidectomy with reconstruction of the posterior wall and the middle ear in a single surgery combines the advantages of both techniques, namely, adequate surgical exposure, eradication of cholesteatoma, and anatomical reconstruction of the middle ear structures.

**Objective:**

To evaluate the surgical results in the management of cholesteatoma through the technique of open mastoidectomy with reconstruction of the posterior wall and the middle ear in a single surgery.

**Methods:**

Prospective analytical observational study conducted between 2009 and 2012 with patients undergoing this surgical technique in the Hospital Universitario del Valle [University Hospital of Valle], performing preoperative clinical monitoring and quarterly postoperative tomography with previous assessments of hearing and pre- and postoperative audiometry.

**Results:**

Forty-five patients were studied. Mean postoperative follow-up was 28 months. Surgical success was achieved in 93.3% of patients, as measured by clinical and radiological follow-up. Hearing preservation was found after reconstruction of the hearing mechanism, based on measured audiometry, i.e., pure-tone average (PTA), using the statistical test for paired samples between preoperative and postoperative PTA. (95%CI -1.47-12.15). Residual cholesteatoma was present in 6.6% of cases; three to four times lower than the rate reported in the literature.

**Conclusions:**

This type of surgery can be considered a successful technique in the treatment of cholesteatoma in selected cases.

## Abstract (es)

**Introducción:**

El manejo del colesteatoma es quirúrgico. Se puede optar por una técnica cerrada (mastoidectomía simple) o técnica abierta (mastoidectomía radical). La mastoidectomía abierta con reconstrucción de la pared posterosuperior y reconstrucción del oído medio en un solo tiempo quirúrgico reúne las bondades de ambas técnicas: una adecuada exposición quirúrgica, erradicación del colesteatoma y reconstrucción anatómica de las estructuras del oído medio.

**Objetivo:**

Evaluar los resultados quirúrgicos en el manejo del colesteatoma con la técnica de mastoidectomía abierta con reconstrucción de la pared posterosuperior y el oído medio en un solo tiempo quirúrgico.

**Métodos:**

Estudio analítico observacional prospectivo realizado entre 2009-2012 con pacientes sometidos a esta técnica quirúrgica en el Hospital Universitario del Valle, realizándose un seguimiento clínico preoperatorio y postoperatorio trimestral, y evaluaciones previas con tomografía de oído y audiometría pre y postoperatoria.

**Resultados:**

Se estudiaron 45 pacientes. La media de seguimiento postoperatorio fue de 28 meses. Se alcanzó éxito quirúrgico en el 93.3% de los pacientes medidos por el seguimiento clínico y radiológico. Se encontró preservación de la audición después de la reconstrucción del mecanismo auditivo, medido con audiometría basada en el promedio tonal auditivo, mediante la prueba estadística para muestras pareadas entre la PTA preoperatoria y postoperatoria. (IC95% -1.47-12.15). Se presentó colesteatoma residual en el 6.6%, tres a cuatro veces menor al reportado en la literatura.

**Conclusiones:**

Este tipo de cirugía se puede considerar una técnica exitosa en el tratamiento de colesteatoma en casos seleccionados.

## Introduction

Chronic otitis media is an inflammatory disease of the middle ear that lasts more than three months. When associated with cholesteatoma, it is characterized by the presence of keratinized stratified squamous epithelium within the cavities of the middle ear. The incidence of cholesteatoma is reported to be between 3.0 and 12.6 cases per 100,000 inhabitants 1. The incidence of congenital cholesteatoma is 0.12 per 100,000 inhabitants in the United States. Recently, strains of *Pseudomonas aeruginosa* have been reported that are capable of producing biofilms, making them highly resistant to antimicrobial therapy. These findings suggest a role for bacterial biofilm in cholesteatoma pathogenesis 2 ^-^ 4. 

Cholesteatoma is managed surgically. The objectives of surgical management of cholesteatomatous chronic otitis media include eradicating the disease, isolating the middle ear from the exterior (anatomically) or obtaining a cavity in the middle ear that is aerated and lined by a viable mucoperiosteum with a stable, fine, and mobile eardrum in a good position. In addition, the recovery or preservation of hearing (functional) by reconstructing the tympanic membrane and mechanism of sound transmission is sought. One can opt for a closed technique with reconstruction of the middle ear or an open technique with meatoconchoplasty. At present, it is uncertain which technique to use, as the closed technique (canal wall up, CWU) with reconstruction preserves the posterior wall of the external auditory canal (EAC) and in the same surgery achieves restoration of anatomy but has limited effectiveness in eradicating cholesteatoma 5. The open technique plus meatoconchoplasty, in which the posterior wall of the EAC is removed, may completely eradicate the infection due to better exposure of ear structures. However, it fails to restore anatomical configuration and hearing mechanism in the same surgery 6 ^,^ 7. To resolve this dichotomy, modified surgical techniques have been developed such as the open technique (canal wall down, CWD) with reconstruction, which removes the posterior wall of the EAC and subsequently reconstructs it along with the middle ear 8 ^,^ 9. There is still a lack of studies that clearly demonstrate which technique is preferable 2 ^,^ 5 ^-^ 9. 

## Materials and Methods

**Figure 1.**
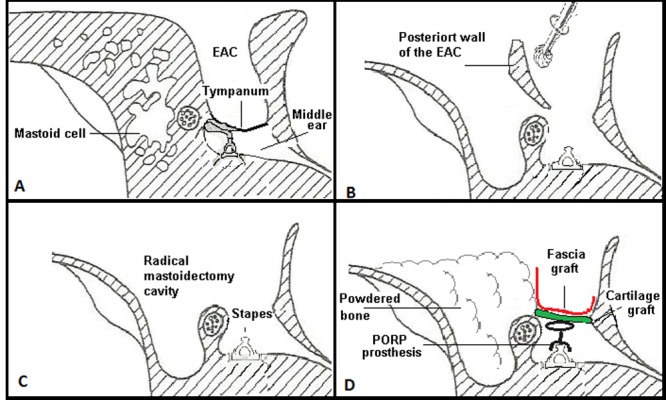
Diagram of open mastoidectomy (CWU) with reconstruction of the posterosuperior wall and middle ear in one surgery. A. One retro-auricular incision is made. B. Mastoid approach with traditional milling. The posterior wall of the EAC was milled to completely expose the facial recess, tympanic sinus, and hypotympanum. C. Milling of the COG (epitympanic ridge dividing the anterior tympanum from the posterior) in the epitympanum until completely exposed (exposure of the anterior and posterior epitympanum). Cholesteatoma was resected, and all spaces were cleaned. D. Mastoid occlusion was performed with powdered bone, cartilage, muscle, and/or temporal fascia. For reconstruction of the ossicular chain, autologous material or titanium prosthesis was used (PORP or TORP)

**Figure 2.**
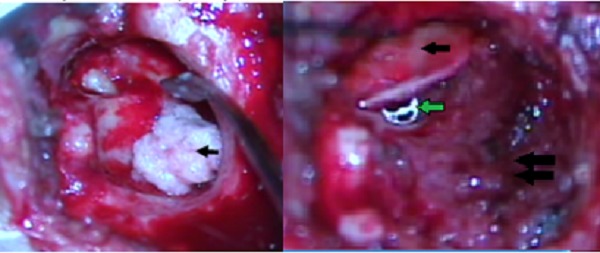
A. Open mastoidectomy with immediate reconstruction. Mastoid obliteration and reconstruction of the posterior wall of the EAC using powdered bone (arrow). B. Open mastoidectomy with immediate reconstruction. Cartilage graft for tympanoplasty (black arrow), titanium TORP prosthesis below the graft (green arrow), posterosuperior wall of the EAC reconstructed with powdered bone (double black arrow)

This paper describes a prospective observational study, using as a sample patients seen at the ear clinic of the Otology and Neuro-Otology service of the University Hospital of Valle in the period between 2009 and 2012. Men and women between 5 and 80 years old diagnosed with cholesteatomatous chronic otomastoiditis who underwent open mastoidectomy with reconstruction in the same surgery were included (Fig. 1A, B, C, D and Fig. 2). Patients for whom surgery was unable to completely remove the cholesteatoma at the first intervention and patients with systemic diseases that contraindicated surgery (according to anesthesiology criteria) were excluded. Postoperative follow-up was conducted at 3, 6 and 12 months, assessing symptoms and findings upon physical examination. Audiometry was tested before surgery, and imaging studies, such as simple scanning (CT) of the middle ear with coronal and axial slices, were conducted. Audiometry was interpreted based on the auditory pure tone average (PTA), which is the average in decibels (dB) at which a patient perceives pure tones at frequencies of 500, 1,000 and 2,000 Hz. Postoperative audiometry was conducted at three months. Statistical analysis of the auditory difference before and after surgery with the paired samples statistic was performed in SPSS. Postoperative MRI ECO-SPIN was requested within a year. The chi-squared test (Χ^2)^ was performed in STATA^®^ 11 to examine the correlation of variables. 

## Results

Forty-five patients (45 ears) were analyzed. The average age of the patients was 32 years old, and 60% were women.

Symptoms and the otoscopic and scanographic findings at presurgical evaluation are described in Table 1. Scanographic findings suggesting cholesteatoma were not detected in 18% (8) of the patients. 

**Table 1.**
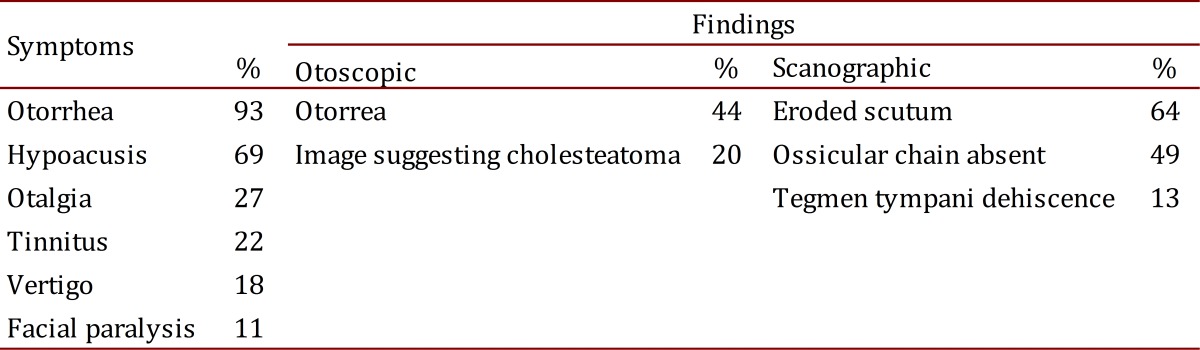
Symptoms, otoscopic and scanographic findings at presurgical evaluation.

The mean time between completing diagnosis and surgery was eight months. During surgery, 57% of patients were found to have a very long cholesteatoma that surpassed the tympanic cavity, which means a very advanced stage of the disease. None had cerebrospinal fluid fistula (Table 2).

**Table 2.**
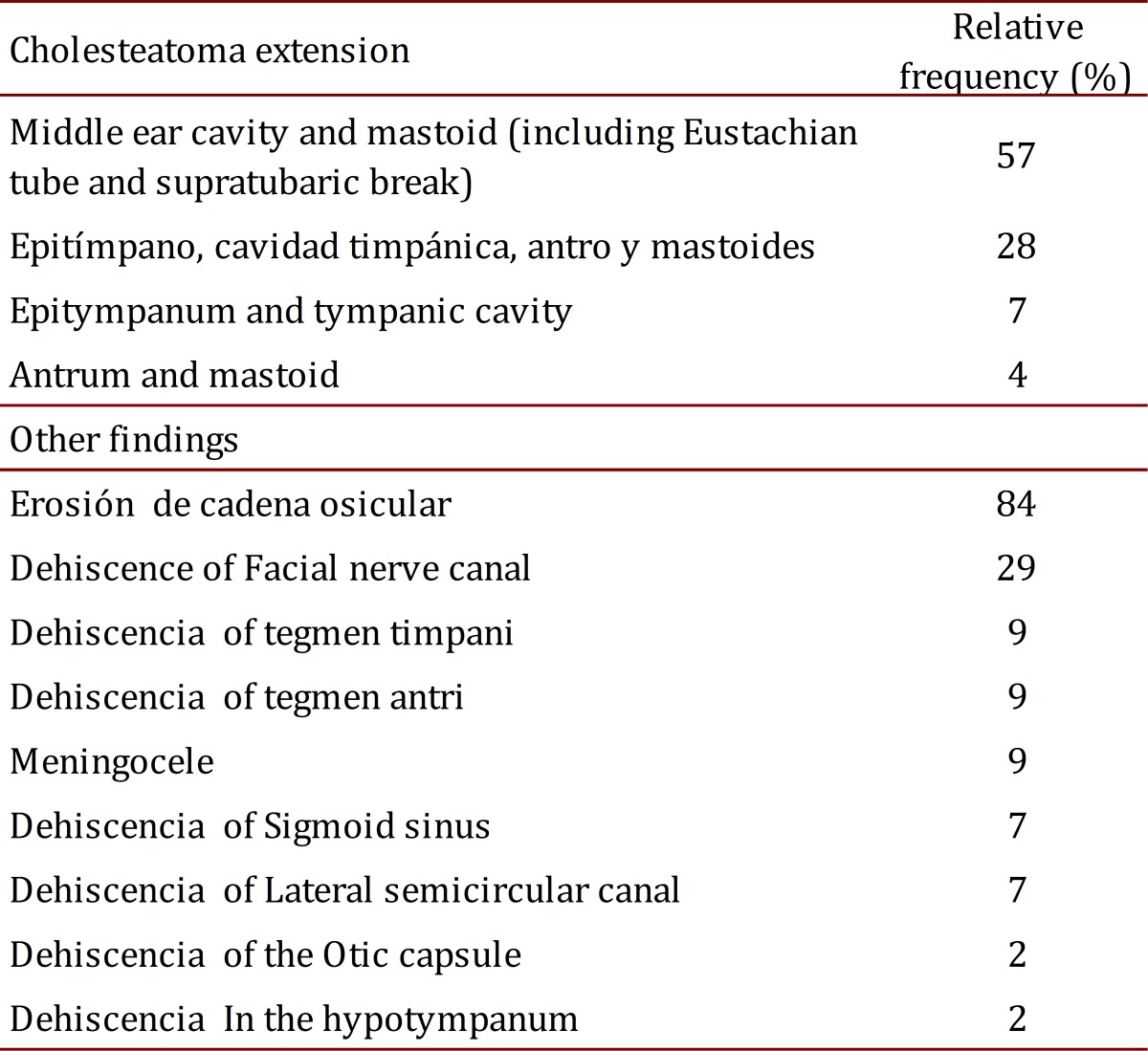
Intraoperative findings of the middle ear.

Erosion of the ossicular chain was most common, observed in 84% of cases, a figure that does not correspond to a scanographic review (49%), with a Kappa value of 0.29 (95% CI: 0.59-0.09), indicating low correlation between the two diagnostic methods. 

Postoperative monitoring was conducted at a minimum of 3 time points, at 3, 6 and 12 months (Table 3). It was considered that some patients had symptoms prior to surgery such as vertigo and facial paralysis, and these symptoms were not noted as positive in the control if there were no postoperative changes. One patient presented EAC granuloma at the 3 month time point, which was then successfully resected under local anesthesia. None had facial paralysis, and only one patient had infection of the surgical wound before the third month, which was satisfactorily resolved with medical treatment. 

**Table 3.**
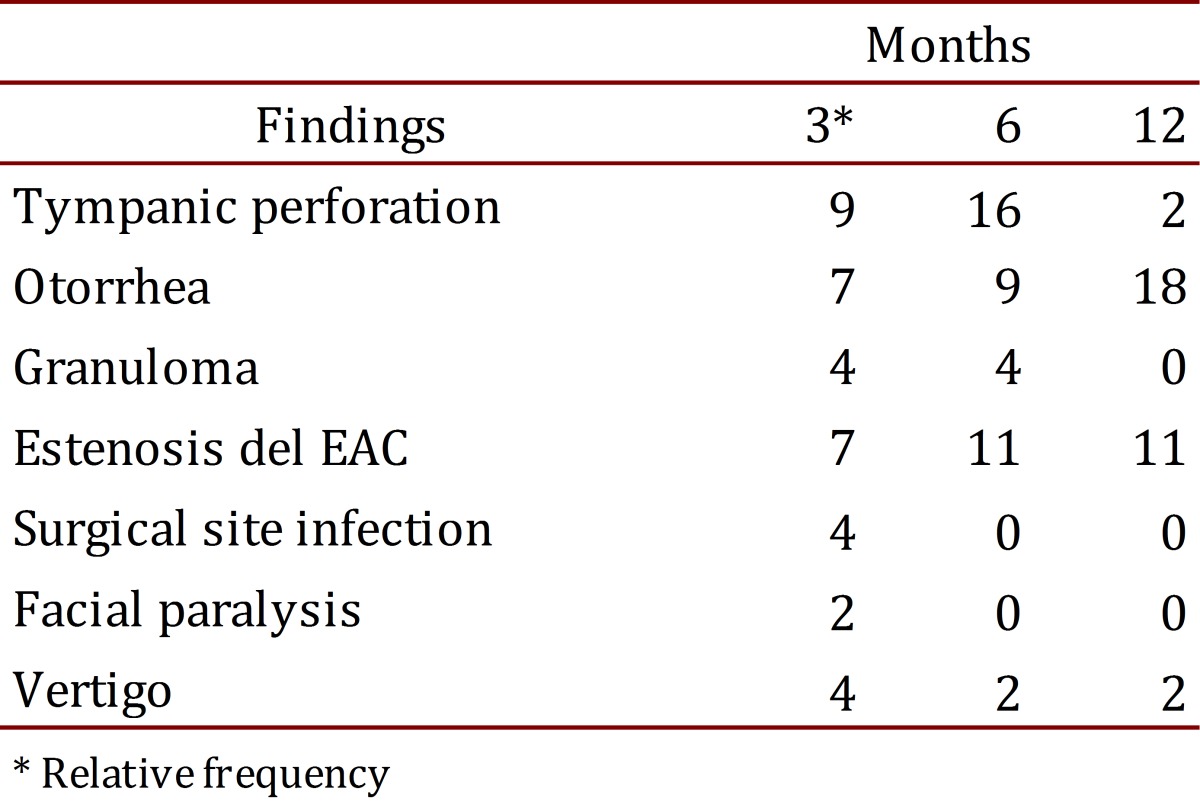
Findings of the postoperative follow-up at 3, 6 and 12 months.

At the 12 month time point, the most common complication continued to be tympanic perforation (20%), followed by otorrhea (18%) and EAC stenosis (11%). A dry ear with signs of adequate healing was achieved in 82% of patients. 

Magnetic resonance imaging (MRI) with the diffusion technique is a valuable tool for monitoring the occurrence of residual/recurrent cholesteatoma to reduce costs and avoid unnecessary revision surgery 10 ^,^ 11. Follow-up with postoperative magnetic resonance was not possible in all cases due to the high cost of the exam and difficulties for health authorities to authorize the exam and cover the cost. MRI could only be performed on four patients (8.8%). 

### Audiological results

Of the 45 patients included in the study, only 27 underwent reconstruction of the entire mechanism of sound transmission, i.e., tympanoplasty with reconstruction of the ossicular chain (11 patients with ossicular reconstruction with autologous tissue and 16 with prosthetic titanium). Reconstruction was not performed in other cases for various reasons, including severe to profound hearing loss (hypoacusis). 

In several cases, postoperative audiometry is not reported in the clinical history, or EAC stenosis was not performed before or after surgery.

The auditory difference before and after surgery was evaluated only in patients with tympanoplasty with reconstruction of the ossicular chain. No differences were found between the audiological condition before and after reconstruction of the hearing mechanism (hearing preservation) in patients undergoing tympanoplasty with reconstruction of the chain (ossiculoplasty). Hearing was measured by audiometry based on the average auditory tone (PTA) through the paired samples statistical test between preoperative PTA and postoperative PTA. (95%CI -1.47-12.15). It was confirmed through this test that there were no statistically significant differences between pre- and postoperative PTA in patients with ossiculoplasty.

### Cholesteatoma recurrence

Cholesteatoma recurrence was confirmed through a second surgical intervention in three patients (6.6%), suspected by MRI in one case and by clinical follow-up (persistence of symptoms) in two cases. 

## Discussion

Surgical management of cholesteatoma and reconstruction of the ear in a single surgery is a highly successful procedure for the total eradication of cholesteatoma. In this series, total elimination of the disease was achieved in 93% of patients undergoing this intervention. Residual cholesteatoma was evident in only 6.6% of subjects. This outcome was highly satisfactory compared to the long-term results reported by Wilson 12. The procedure has the benefit of a small, self-cleaning, impermeable, and properly healed cavity in 85% of patients at one year of follow-up, without detriment to hearing function. It constitutes a significant contribution to quality of life and extends the possibility of rehabilitating the hearing organ. 

Today, cholesteatoma is not a mortality issue; it is a morbidity problem. Deterioration of quality of life as a result of hearing loss secondary to the disease and sequelae associated with radical operations with extensive mastoidectomy cavities (very effective for controlling cholesteatoma and intracranial complications), which require lifelong care from moisture and cleaning by trained personnel and which suffer a high risk of colonization by fungi and bacteria, make these issues a priority 13. Since the mid-1960s, several authors such as Portman, House, and Lempert, with the advent of new technologies such as the operating microscope, milling motors, and microsurgical instruments, introduced the concept of the "closed technique" for managing cholesteatoma. This technique entailed a surgical procedure for removing cholesteatoma where the posterior wall of the EAC is preserved or reconstructed to leave a small self-cleaning, impermeable cavity similar to the EAC, followed by the first attempts to reconstruct the tympanic membrane and the ossicular chain to restore hearing. Of course, this type of surgery had indicated advantages, but there was still doubt as to whether it completely eradicated the cholesteatoma and whether the risk of brain abscess and purulent meningitis was eliminated. To control this uncertainty, a second look became necessary a year after the first surgery to check hearing and confirm the success or failure of the first intervention. 

Published experiences on cholesteatoma recurrence managed with the closed technique range from 22% to 40%, and although the initial results were good, they were not sustained over time 12. The closed technique for managing cholesteatoma with a second look generated much controversy versus management with the traditional open technique of radical operation and its many modifications. The open technique provides better control of cholesteatoma recurrence with recurrence indices of 15% but has greater associated morbidity. The closed technique has better initial hearing results and less morbidity but a higher recurrence rate, up to 40% 12.

Having the opportunity at present to perform follow-up imaging of cholesteatoma with magnetic resonance imaging (MRI) with the diffusion technique without requiring a second look operation has led to resurgence in interest in managing cholesteatoma with the closed technique. 

In this study, 45 ears with cholesteatomatous chronic otitis were treated with the open technique (CWD) with reconstruction at a single surgery, resulting in 93.3% success for control of cholesteatoma and only 6.6% recurrence, well below the rate reported in the literature 11. 

The impact of this procedure on quality of life is such that self-cleaning and impermeable cavities are obtained consistently, and in our experience, only 10% required management with the open technique when certainty of total eradication was not achieved. Although hearing results are not better than the results reported by other series, they are comparable to the results reported internationally by authors such as Schuknecht, Colletti, and Goldenberg, where the percentage of cases with air vs. bone gap lower than 20 dB for type II tympanoplasty is between 40% and 60% and for type III tympanoplasty is between 28 and 58% 14 ^-^ 17. The closed technique provides the advantage of leaving an impermeable, small, self-cleaning cavity susceptible to audiological self-rehabilitation with digital hearing aids tailored by air. It is an economical method that is contraindicated in open cavities due to the continued need for external ventilation and expansion of the external auditory meatus, leaving expensive bone conduction implants as the only option for the rehabilitation of such patients, which are not free of morbidity. 

There is still much room for improvement in terms of reconstruction of the tympanum-ossicular system to provide better hearing closer to the natural experience of sound and to comprehensively rehabilitate patients with cholesteatomatous chronic otitis in a single surgery. 

## Conclusions

Open mastoidectomy with reconstruction performed in a single surgical procedure can be considered a highly successful (93%) technique for treating cholesteatoma, with recurrence between three and four times lower than reported in the literature as well as preservation of hearing. This technique prevents the cavity from filling with scabs, fungus, and bacterial infections repeatedly, and it further benefits the patient by avoiding a second surgery and benefits the institution through decreased costs. However, it requires a highly individualized approach and should consider the anatomical, clinical, and social factors of the patient to determine the most successful surgical treatment. The surgeon's judgment is very important for making a final determination. Lack of health awareness, low socioeconomic status, and lack of adequate access to health care lead to poor results and inadequate follow-up in the postoperative period. For patients who are difficult to follow, have extensive disease, or have disease in an ear with severe to profound hearing loss, we prefer to use an open technique with meatoconchoplasty. 
